# Lithium Superionic Conduction in BH_4_‐Substituted Thiophosphate Solid Electrolytes

**DOI:** 10.1002/advs.202204942

**Published:** 2022-12-11

**Authors:** Yong‐Jin Jang, Hyungeun Seo, Young‐Su Lee, Sora Kang, Woosuk Cho, Young Whan Cho, Jae‐Hun Kim

**Affiliations:** ^1^ School of Materials Science and Engineering Kookmin University Seoul 02707 Republic of Korea; ^2^ Energy Materials Research Center Korea Institute of Science and Technology Seoul 02792 Republic of Korea; ^3^ Advanced Batteries Research Center Korea Electronics Technology Institute Seongnam Gyeonggi‐do 13509 Republic of Korea

**Keywords:** all‐solid‐state batteries, argyrodite, lithium borohydride, solid electrolyte, thiophosphate

## Abstract

Compared with conventional liquid electrolytes, solid electrolytes can better improve the safety properties and achieve high‐energy‐density Li‐ion batteries. Sulfide‐based solid electrolytes have attracted significant attention owing to their high ionic conductivities, which are comparable to those of their liquid counterparts. Among them, Li thiophosphates, including Li‐argyrodites, are widely studied. In this study, Li thiophosphate solid electrolytes containing BH_4_^−^ anions are prepared via a simple and fast milling method even without heat treatment. The synthesized materials exhibit a high ionic conductivity of up to 11 mS cm^−1^ at 25 °C, which is much higher than reported values. To elucidate the mechanism behind, the thiophosphate local structure, whose effect on the ionic conductivity remains unclear to date, is investigated. Raman and solid‐state NMR spectroscopies are performed to identify the thiophosphate local structure in the sulfide samples. Based on the analysis results, the ratios of the different thiophosphate units in the prepared electrolyte samples are determined. It is found that the thiophosphate local structure can be varied by changing the amount of LiBH_4_ and the milling conditions, which significantly impact the ionic conductivity. The all‐solid‐state cell with the prepared solid electrolyte exhibits superior cycle and rate performances.

## Graphical abstract

A BH_4_‐substituted thiophosphate material including an argyrodite phase is synthesized by simple two‐step milling process, which exhibits a high Li ionic conductivity of 11 mS cm^−1^ at 25 °C. To investigate the mechanism behind, solid‐state NMR spectra are analyzed and the effect of the thiophosphate local structure on the ionic conductivity is revealed.

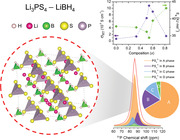

## 1 Introduction

In recent years, the use of electric vehicles has accelerated worldwide, and Li‐ion batteries (LIBs) continue to dominate the electrochemical power source market. In the meantime, the demand for high‐energy‐density LIBs is rapidly growing, giving rise to safety concerns about such batteries. One of the major strategies to meet the demands for high energy density and safety is to replace the flammable and volatile liquid electrolytes with solid electrolytes in the current LIBs. Inorganic solid electrolytes with a wide electrochemical stability window of up to 5 V can facilitate the use of high‐capacity Li metal anodes or anode‐free systems by reducing the formation of dendritic Li growth.^[^ ^1^ ^]^ Additionally, solid electrolytes can remarkably enhance the safety of LIBs, because they are relatively stable compared to organic liquid electrolytes in the event of thermal runaway.

For several decades, all‐solid‐state batteries (ASSBs) with solid electrolytes have been actively investigated by many researchers. The low ionic conductivities of the solid electrolytes used in the ASSBs as well as the interface incompatibility between the electrode active materials and the solid electrolytes are the major problems associated with the ASSBs. These problems were difficult to address, and consequently, slowed the progress of the field. The ionic conductivity issues, however, have been advanced in the 2010s with demonstration of sulfide‐based Li superionic conductors such as Li_10_GeP_2_S_12_, whose conductivities are equal or superior to those of liquid electrolytes. Various sulfide‐based solid electrolytes reportedly exhibit high ionic conductivities over 1 mS cm^−1^ at room temperature.^[^ ^2^ ^]^ Specifically, a type of thio‐LISICON (Li_10_GeP_2_S_12_) reportedly showed a high ionic conductivity of 1.2 × 10^−2^ S cm^−1^.^[^ ^3^ ^]^ In addition, a heat‐treated Li_2_S‐P_2_S_5_ glass‐ceramic material demonstrated a high conductivity of 1.7 × 10^−2^ S cm^−1^.^[^ ^4^ ^]^ Further, argyrodite‐type materials, e.g., Li_6_PS_5_X (X = Cl, Br, and I), which are a main category of the sulfide solid electrolytes, exhibit high ionic conductivities of 10^−2^ to 10^−3^ S cm^−1^.^[^ ^5^ ^]^

Lithium borohydride (LiBH_4_) is considered an attractive solid electrolyte because of its high conductivity at high temperatures and good compatibility with Li metal.^[^ ^6^ ^]^ Although LiBH_4_ is itself considered a viable candidate, some researchers tried to incorporate this material into Li_2_S‐P_2_S_5_ glass and argyrodite‐type materials to further improve the ionic conductivity. Yamauchi et al. reported the synthesis and characterization of solid electrolytes with a composition of (100−*x*)(0.75Li_2_S−0.25P_2_S_5_)·*x*LiBH_4_ via a two‐step ball milling.^[^ ^7^ ^]^ For the composition with *x* ≤ 33, glass‐ceramic materials, including PS_4_^3−^ and BH_4_^−^ anions, were prepared, and an ionic conductivity of 1.6 × 10^−3^ S cm^−1^ was obtained at 25 °C. In the following study, a crystalline material was synthesized with a composition corresponding to *x* ≥ 43.^[^ ^8^ ^]^ The crystalline phase was identified as an argyrodite structure with a composition of Li_6_PS_5_X, where X was replaced with the BH_4_^−^ ions. In other studies, Cl or I in the argyrodite crystal was partially or fully replaced with borohydride ions.^[^ ^9^ ^]^ The borohydride‐substituted materials exhibited a higher stability with Li metal and enhanced ionic conductivities than did Li_6_PS_5_X (X = Cl and I). However, the ionic conductivity was still low, and the effect of the local structure of the thiophosphate on the ionic conductivity remains hitherto underexplored.

In this study, we prepared borohydride‐substituted Li thiophosphate materials with a composition of (1−*x*)Li_3_PS_4_·2*x*LiBH_4_ by a two‐step milling process without heat treatment. As the amount of LiBH_4_ was increased, the formation of an argyrodite‐type phase, including BH_4_^−^ anions, was identified, and a high ionic conductivity of up to 11 mS cm^−1^ was measured at room temperature. To examine the influence of thiophosphate local structure on the ionic conductivity of the borohydride‐substituted solid electrolytes, Raman and solid‐state magic angle spinning NMR (MAS NMR) spectroscopic analyses were performed. Based on the analyses results, we determined the ratios of different thiophosphate units and the location of the main PS_4_^3−^ units in the prepared electrolyte samples. The thiophosphate local structure of the borohydride‐substituted materials could be varied by changing the amount of LiBH_4_ and milling conditions, which significantly influenced the ionic conductivity. Eventually, the correlation between the thiophosphate local structure and the ionic conductivity was examined, and the effect of the local structure was demonstrated.

## 2 Results and Discussion

**Figure 1:**
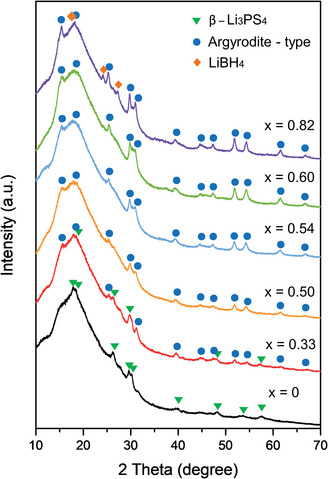
XRD patterns of the prepared solid electrolyte samples with the composition of (1−*x*)Li_3_PS_4_·2*x*LiBH_4_.

In this study, a high rotation speed and large zirconia balls for mechanical milling were adopted (see the Experimental Section). To deduce the influence of milling conditions on the thiophosphate local structure, we also prepared samples under different milling conditions (relatively low rotation speeds and small balls; reference condition).^[^ ^8^ ^]^ Hereafter, “10mm” (or no mark) indicates the samples prepared using 10 mm balls with a high rotation speed, and “5mm” refers to the samples synthesized under the reference condition. **Figure** **1** shows the X‐ray diffraction (XRD) patterns of the solid electrolyte samples prepared by the two‐step mechanical milling. The compositions can be expressed as (1−*x*)Li_3_PS_4_·2*x*LiBH_4_, where (1−*x*):2*x* is the molar ratio of Li_3_PS_4_:LiBH_4_ in the samples. After the first‐step milling with Li_2_S and P_2_S_5_ powders at a molar ratio of 3:1 (*x* = 0), only the diffraction peaks of the *β*‐Li_3_PS_4_ phase with *Pnma* space group were observed.^[^ ^10^ ^]^ Although a broad background was detected suggesting the formation of amorphous phases, the sample prepared by the first‐step milling was denoted as Li_3_PS_4_. After the two‐step milling, the final samples were obtained (hereafter, referred to as (1−*x*)Li_3_PS_4_·2*x*LiBH_4_). When the content of LiBH_4_ was increased in the samples, the patterns showed the *β*‐Li_3_PS_4_ phase and a crystalline phase, which is not indexed in the International Committee on Diffraction Data. However, the unknown phase can be indexed to an argyrodite‐type phase according to the previous reports.^[^ ^8^, ^11^ ^]^ The argyrodite‐type phase, which originates when the sulfur sites in Li_7_PS_6_ are substituted with halides, consists of PS_4_^3−^ anions with Li_2_S and LiX (X = Cl, Br, I).^[^ ^1^, ^12^ ^]^ Therefore, the crystalline phase is considered to be an argyrodite‐type phase in which the sulfur sites are replaced by BH_4_^−^ ions. The sample with *x* = 0.33 exhibited both the phases, indicating that a part of *β*‐Li_3_PS_4_ did not react. The samples with *x* = 0.50 to *x* = 0.60 showed the argyrodite phase only. When *x* increased to 0.82, the diffraction peaks of LiBH_4_ were observed as well, revealing that a certain amount of LiBH_4_ remained unreacted during the mechanical milling. The XRD analysis showed that an optimum composition can exist to form the argyrodite‐type phase including BH_4_^−^. The XRD patterns of the *x* = 0 (5mm) and *x* = 0.54 (5mm) samples prepared under the reference condition are shown in Figure S1 (Supporting Information). The *x* = 0 (5mm) sample showed amorphous backgrounds with weak diffraction peaks for Li_2_S. Under this condition, *β*‐Li_3_PS_4_ was not produced, and a certain amount of Li_2_S remained unreacted. These results were obtained because the milling energy to form *β*‐Li_3_PS_4_ was insufficient due to the lower rotation speed of the mill and the smaller ZrO_2_ balls. The pattern of the *x* = 0.54 (5mm) sample indicated that the argyrodite‐type phase can be well created like that in the *x* = 0.54 (10mm) sample. Further, differential scanning calorimetry (DSC) was employed to assess the thermal properties of the prepared samples, and the resulting profiles are shown in Figure S2 (Supporting Information, please see related text). A peak corresponding to the phase transition of the unreacted LiBH_4_ was observed. The morphology of the *x* = 0.54 (10mm) sample was characterized by electron microscopies and the results with explanation are presented in Figure S3 in the Supporting Information.

**Figure 2:**
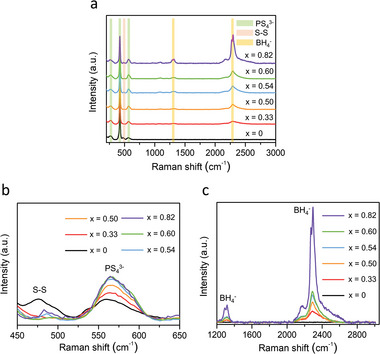
a) Raman spectra of the prepared solid electrolyte samples with the composition of (1−*x*)Li_3_PS_4_·2*x*LiBH_4_ and b,c) enlarged Raman spectra at specific regions.

**Figure** **2**a shows the Raman spectra of the prepared solid electrolyte samples with the composition of (1−*x*)Li_3_PS_4_·2*x*LiBH_4_. All the samples showed the vibrational modes of PS_4_^3−^ anions at 272, 423, and 572 cm^−1^.^[^ ^13^ ^]^ After the addition of LiBH_4_ into the Li_3_PS_4_ sample via the second‐step milling, the signals for the S—S bond vibrations at 473 cm^−1^, which was visible for the Li_3_PS_4_ sample (*x* = 0), disappeared.^[^ ^13^ ^]^ Moreover, the BH_4_^−^ anion (at 1302 and 2299 cm^−1^) vibrations appeared.^[^ ^8^, ^14^ ^]^ These results demonstrated that the new phase was created by the incorporation of LiBH_4_, which was attributed to the argyrodite‐type phase identified in the XRD analysis. To investigate the phase changes with the increasing *x* values, the Raman spectra were enlarged between 450 and 650 cm^−1^ as shown in Figure 2b. As the amount of LiBH_4_ increased up to *x* = 0.54, the peak intensities at 572 cm^−1^ for the PS_4_^3−^ anions gradually increased. When *x* was more than 0.54, the intensity remained similar. The S—S bond peak at 473 cm^−1^ almost disappeared with the incorporation of LiBH_4_. Figure 2c compares the Raman spectra between 1200 and 2700 cm^−1^. With increasing the amount of LiBH_4_ up to *x* = 0.54, the peak intensities of the BH_4_^−^ anions (at 1302 and 2299 cm^−1^) progressively increased. Especially, for the *x* = 0.82 sample, the peak intensities of the BH_4_^−^ anions were extremely high, because it contained a significant amount of free LiBH_4_. The shapes of the Raman profiles at 1302 and 2299 cm^−1^ were similar to that of the pure LiBH_4_ powder (see Figure S4, Supporting Information).^[^ ^8^, ^14^ ^]^ Therefore, it was confirmed again that a large amount of unreacted LiBH_4_ remained in the *x* = 0.82 sample. Based on the results of the XRD, DSC, and Raman analyses, it was noted that the Li_3_PS_4_ sample, including the PS_4_^3−^ anions, were co‐crystallized with the BH_4_^−^ anions to form the argyrodite‐type phase via the second‐step milling process with the increasing amount of LiBH_4_ up to *x* = 0.54. At this point, the formation of the argyrodite‐type phase was saturated, and the excess LiBH_4_ remained unreacted in the samples.

The Raman spectra of the *x* = 0 (5mm) and *x* = 0.54 (5mm) samples prepared under the reference condition are shown in Figure S5a in the Supporting Information. The spectral shapes were similar to those of the “10mm” samples. In the *x* = 0 (5mm) sample, a peak at 2550 cm^−1^ was detected, which can be assigned to the vibrational mode of Li_2_S (see Figure S4, Supporting Information), implying that this sample contained a small amount of unreacted Li_2_S, consistent with the aforementioned XRD analysis result. To compare the samples milled under different conditions, the profiles between 370 and 470 cm^−1^ were overlapped (Figure S5b,c, Supporting Information). For the “5mm” samples, a shoulder at 385 cm^−1^ was observed, which can be attributed to the vibration mode of the P_2_S_6_^4−^ units.^[^ ^15^ ^]^ The existence of the P_2_S_6_^4−^ units can significantly influence the ionic conductivity of the electrolyte samples, as discussed later.

**Figure 3:**
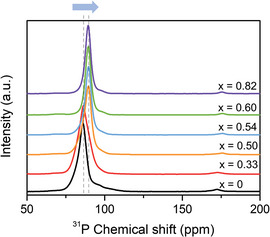
^31^P solid‐state MAS NMR spectra of the prepared solid electrolyte samples with the composition of (1−*x*)Li_3_PS_4_·2*x*LiBH_4_.

**Figure** **3** shows the ^31^P solid‐state MAS NMR spectra of the prepared samples with the composition of (1−*x*)Li_3_PS_4_·2*x*LiBH_4_. The main peak for the *x* = 0 sample was observed at 86.1 ppm and can be ascribed to the PS_4_^3−^ anion units in the *β*‐Li_3_PS_4_ phase,^[^ ^16^ ^]^ which was identified in the XRD analysis. When the amount of LiBH_4_ in the sample was increased to *x* = 0.50, the main peak position of each sample slightly shifted toward a higher frequency. Above this point (*x* = 0.50), the position remained almost the same at 89.5 ppm. In the XRD patterns, only the argyrodite‐type phase was detected at the compositions of *x* = 0.50, 0.54, and 0.60, indicating that the main peak at 89.5 ppm can be attributed to the PS_4_^3−^ units in the argyrodite‐type phase.

The ^31^P MAS NMR spectra of the *x* = 0 (5mm) and *x* = 0.54 (5mm) samples prepared under the reference condition are shown in Figure S6a in the Supporting Information. At ≈106–108 ppm, the “5mm” samples exhibited different peaks, which can be attributed to the P_2_S_6_^4−^ units.^[^ ^14^, ^15^ ^]^ The peaks were not observed in the “10mm” samples (see Figure S6b,c, Supporting Information). For the *x* = 0 (5mm) sample, the main peak was located at 83.4 ppm, which can be ascribed to the amorphous Li_3_PS_4_ phase based on the results of the XRD and Raman analyses. From these results, it can be concluded that the main peak is related to the PS_4_^3−^ units in the amorphous phase.^[^ ^16a^ ^]^ After the addition of LiBH_4_, the *x* = 0.54 (5mm) sample showed the main peak position at 89.5 ppm, similar to that observed for the *x* = 0.54 (10mm) sample. This result well corresponds to the XRD results, because both the *x* = 0.54 (5mm) and *x* = 0.54 (10mm) samples exhibited diffraction peaks corresponding to the argyrodite‐type phase. In both the cases, the second‐step milling resulted in the formation of the argyrodite‐type phase including the BH_4_^−^ anions, through reactions with either the PS_4_^3−^ units in the amorphous Li_3_PS_4_ phase or in the *β*‐Li_3_PS_4_ phase.

The ionic conductivities of the solid electrolyte samples were measured at 25 °C using electrochemical impedance spectroscopy (EIS), and the results are shown in **Table** **1**. The Nyquist plots of the samples are depicted in Figure S7 in the Supporting Information. For the “10mm” samples prepared in this study, the ionic conductivity increased with the increasing LiBH_4_ concentration up to *x* = 0.54, where the conductivity was 1.1 × 10^−2^ S cm^−1^. This is large value and can exceed those of the conventional nonaqueous liquid electrolytes used in Li‐ion batteries.^[^ ^4^ ^]^ Furthermore, this conductivity value is much higher than those reported in previous studies on borohydride argyrodite materials.^[^ ^7^, ^8^, ^9^, ^17^ ^]^ This result can be attributed to the thiophosphate local structure of the prepared materials, which is favorable for Li‐ion conduction. The local structure was closely examined as described later. When *x* = 0.60 and 0.82, the conductivity decreased, which can be simply interpreted based on the XRD, Raman, and ^31^P MAS NMR analyses results. The content of the argyrodite‐type phase substituted by the BH_4_^−^ anions in the milled samples can basically determine the ionic conductivity. In the *x* = 0.54 sample, the creation of the argyrodite‐type phase by the second‐step milling was saturated, and the conductivity was the highest. Notably, the argyrodite‐type phase substituted by the BH_4_^−^ anions can play an important role in the enhancement of the ionic conductivity. In the case of the *x* = 0.54 (5mm) sample prepared under the reference condition, the conductivity was found to be 4.8 × 10^−3^ S cm^−1^, which is much lower than that of the *x* = 0.54 (10mm) sample. From the Raman and ^31^P MAS NMR analyses results, the existence of the P_2_S_6_^4−^ anion units in the *x* = 0.54 (5mm) sample was revealed. To elucidate the influence of the composition and synthesis conditions on the ionic conductivity via the formation of different thiophosphate anion units, more elaborate spectroscopic analyses are required.

**Table 1:** Ionic conductivities of the prepared solid electrolyte samples

*x*	Composition	Ionic conductivity [10^−3^ S cm^−1^ at 25 °C]
0 (10mm)	Li_3_PS_4_	0.66
0.33 (10mm)	Li_4_PS_4_(BH_4_)	1.4
0.50 (10mm)	Li_5_PS_4_(BH_4_)_2_	7.0
0.54 (10mm)	Li_5.3_PS_4_(BH_4_)_2.3_	11
0.60 (10mm)	Li_6_PS_4_(BH_4_)_3_	9.0
0.82 (10mm)	Li_12_PS_4_(BH_4_)_9_	1.5
0 (5mm)	Li_3_PS_4_	0.21
0.54 (5mm)	Li_5.3_PS_4_(BH_4_)_2.3_	4.8

The ionic conductivity of *x* = 0.54 (10mm) sample is higher than some other halide‐substituted Li_6−_ *_y_* PS_5−_ *_y_* X_1+_ *_y_*,^[^ ^2^, ^18^ ^]^ and we investigated why the substitution by BH_4_ leads to higher ionic conductivity. Recent experimental and theoretical studies revealed that the distribution of X over the 4*a* and 4*d* Wyckoff sites of *F*‐43*m* space group strongly affects ionic conductivity.^[^ ^18^ ^]^ Generally, a halide ion X prefers the 4*a* site (X@4*a*) at room temperature, but increased occupancy at the 4*d* site (X@4*d*) at high temperature can be frozen to room temperature and this disorder makes Li diffusion pathway more favorable to diffusion. In this regard, the site preference of BH_4_ in the Li_6_PS_5_X structure was assessed by density functional theory (DFT) calculations. For simplicity, we assumed that Li ions fully occupy the 24*g* site for the X@4*a* structure to avoid the complexity of a partial occupancy at the 48*h* site. In the X@4*d* structure, Li ions at the 24*g* site cannot approach closely to the S ions at the 4*a* site. To make a local structure similar to the X@4*a* structure, Li ions were placed at the 24*f* site. The crystal structure is shown in Figure S8 in the Supporting Information. Six Li ions surround a S ion irrespective of the position of S.

First, the lattice parameters of those two structures were compared. The optimized lattice parameters in Figure S9a in the Supporting Information show almost linear relationship with the tabulated ionic radius of X regardless of the Wyckoff position of X.^[^ ^19^ ^]^ We note that the calculated lattice parameters are bigger than the experimental ones due to the hypothetical arrangement of Li ions. On the contrary, the total energy difference between the two structures, Δ*E* = *E*(X@4*d*) − *E*(X@4*a*), in Figure S9b in the Supporting Information demonstrates that Δ*E* of X = BH_4_ deviates from the trend. Again due to the restriction in the Li position, Δ*E* decreases with ionic radius, but in reality, it should increase with ionic radius since a smaller halide ion prefers a site disorder.^[^ ^20^ ^]^ Here, we focus only on whether BH_4_ follows the trend predicted by the ionic radius, not the absolute Δ*E* values. The relatively small Δ*E* of BH_4_ can be ascribed to the directional bonding unique to BH_4_. Within the *F*‐43*m* symmetry, BH_4_ can take two orientations as shown in Figure S10a in the Supporting Information. When Li ions are close to S at the 4*a* site, the reorientation of BH_4_ can greatly reduce Δ*E* from 0.56 to 0.32 eV f.u.^−1^ as shown in Figure S10b in the Supporting Information. This result indicates that BH_4_ anions in the argyrodite structure may exhibit higher degree of disorder over the 4*a* and 4*d* sites thereby enhancing the ionic conductivity.

In addition, bond distance between S and Li again deviates from the trend when BH_4_ is substituted. As shown in Figure S11 in the Supporting Information, the distance between S at the 16*e* site (S in PS_4_) and Li becomes longer than expected whereas S at the 4*a* or 4*d* makes shorter bond with Li. BH_4_ also affects effective charges: PS_4_ and S become less negative since BH_4_ draws more charge than the halide ions (Figure S12, Supporting Information). Overall, the interaction of Li with PS_4_ appears to be weakened by BH_4_, and the reduced charge difference between S and BH_4_ (Figure S12d, Supporting Information) may lead to more uniform Li distribution. While a comprehensive molecular dynamics study is indeed necessary to fully understand the effect of BH_4_ ions in the realistic Li distribution, the current result strongly suggests that BH_4_ ions in the argyrodite structure would result in the Li‐ion distribution and diffusion behavior which are distinct from the halide‐substituted structure.

**Figure 4:**
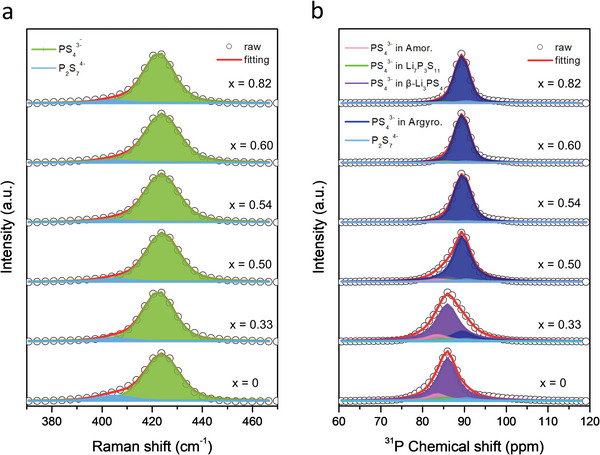
a) Raman spectra with deconvoluted profiles and b) ^31^P solid‐state MAS NMR spectra with deconvoluted profiles of the prepared solid electrolyte samples with the composition of (1−*x*)Li_3_PS_4_·2*x*LiBH_4_.

Dietrich et al. reported the influence of the different thiophosphate units on the ionic conductivity of (Li_2_S)*_x_* (P_2_S_5_)_100−_ *_x_*.^[^ ^15a^ ^]^ The thiophosphate building units, including ortho‐thiophosphate (PS_4_^3−^, monomers), pyro‐thiophosphate (P_2_S_7_^4−^, dimers), and hypo‐thiodiphosphate (P_2_S_6_^4−^, P—P‐dimers), were identified in the deconvoluted ^31^P MAS NMR and Raman spectra. In the synthesis process of this study, the amorphous or *β*‐Li_3_PS_4_ phase was mainly formed after the first‐step milling, which mainly consisted of the PS_4_^3−^ units based on the stoichiometry of the starting material. Then, the second‐step milling led to the formation of the argyrodite‐type phase via reaction with LiBH_4_, and this resulting argyrodite‐type phase was also composed of the PS_4_^3−^ anion units. However, the milled samples can contain other thiophosphate units, such as P_2_S_7_^4−^ and P_2_S_6_^4−^, which may originate from the side reaction or nonstoichiometric reaction during the fabrication process.^[^ ^11^ ^]^ Therefore, to examine the influence of the local structure on the ionic conductivity, it is essential to calculate the ratio among the different thiophosphate units (PS_4_^3−^, P_2_S_7_^4−^, and P_2_S_6_^4−^). To perform the calculations in terms of the composition (*x*) and milling conditions (“5mm” and “10mm”), we first deconvoluted the Raman and ^31^P MAS NMR spectra of the prepared solid electrolyte samples with the composition of (1−*x*)Li_3_PS_4_·2*x*LiBH_4_. The deconvolution results are shown in **Figure** **4**, and the abundance ratios for the thiophosphate units are presented in **Table** **2**.

**Table 2:** Abundance ratios of the thiophosphate units calculated from deconvolution data of the Raman spectra (Voigt product function) and ^31^P solid‐state MAS NMR (Voigt and Lorentzian product function) spectra of the prepared solid electrolyte samples with the composition of (1−*x*)Li_3_PS_4_·2*x*LiBH_4_

	Raman spectra			^31^P solid‐state MAS NMR spectra					
*x*	Peak indexing	PS_4_^3−^ units	P_2_S_7_^4−^ units	Peak indexing	PS_4_^3−^ units in amorphous phase	PS_4_^3−^ units in Li_7_P_3_S_11_	PS_4_^3−^ units in *β*‐Li_3_PS_4_	PS_4_^3−^ units in argyrodite phase	P_2_S_7_^4−^ units
0	Peak (cm^−1^)	422.9	404.9	Peak (ppm)	83.4	85.5	86	‐	90.4
	Area (%)	90.9	9.1	Area (%)	12.2	5.6	77.1	‐	5.1
0.33	Peak (cm^−1^)	423.4	405.2	Peak (ppm)	83.4	85.5	86	89.6	90.4
	Area (%)	94.2	5.8	Area (%)	11.2	3.7	63.9	17.1	4.1
0.50	Peak (cm^−1^)	423.7	405.1	Peak (ppm)	83.5	85.5	86	89.5	90.5
	Area (%)	94.5	5.5	Area (%)	4.5	4.1	7.9	79.4	4.1
0.54	Peak (cm^−1^)	423.5	405.1	Peak (ppm)	83.5	85.4	86.1	89.6	90.4
	Area (%)	95.4	4.6	Area (%)	1.4	2.3	2.6	91.6	2.1
0.60	Peak (cm^−1^)	423.8	405.1	Peak (ppm)	83.5	85.5	85.9	89.4	90.5
	Area (%)	95.5	4.5	Area (%)	1.2	2.1	2.7	91.7	2.3
0.82	Peak (cm^−1^)	422.8	404.7	Peak (ppm)	83.4	85.5	86	89.5	90.4
	Area (%)	94.7	5.3	Area (%)	1.6	2.4	2.2	91.6	2.2

Figure 4a shows the Raman spectra with deconvoluted profiles between 370 and 470 cm^−1^. As shown in Figure 2, the sub‐profiles centered at around 423 cm^−1^ were observed for all the samples. These were assigned to the vibrational mode of the PS_4_^3−^ units, and the sub‐profiles at ≈405 cm^−1^ were attributed to the P_2_S_7_^4−^ units. According to previous studies, the Raman spectra could be deconvoluted to the two thiophosphate units.^[^ ^15^, ^21^ ^]^ The abundance ratio was calculated based on the area of each sub‐profile (Table 2). After the first‐step milling (*x* = 0), the abundance ratios of the PS_4_^3−^ and P_2_S_7_^4−^ units were found to be 90.9% and 9.1%, respectively. After the second‐step milling with LiBH_4_ (*x* = 0.33–0.82), the areal ratios for the PS_4_^3−^ units increased to 94.2–95.5%, while those of the P_2_S_7_^4−^ units decreased to 4.5–5.8%. This indicates that the creation of the argyrodite‐type phase containing the BH_4_^−^ anions reduces the production of the nonstoichiometric P_2_S_7_^4−^ units.

The deconvoluted results of the Raman spectra enabled us just to distinguish the thiophosphate units such as PS_4_^3−^ and P_2_S_7_^4−^. According to previous studies, the deconvolution of the ^31^P solid‐state MAS NMR spectra can be useful to identify where the PS_4_^3−^ units exist because of the analytical sensitivity of this technique.^[^ ^22^ ^]^ Figure 4b shows the ^31^P MAS NMR spectra with deconvoluted sub‐profiles. Gobet et al. reported different chemical shifts in the ^31^P MAS NMR spectra of the PS_4_^3−^ units in various environments such as amorphous and crystalline Li_3_PS_4_.^[^ ^16a^ ^]^ Notably, the P_2_S_7_^4−^ units can co‐exist with the PS_4_^3−^ units in the Li_7_P_3_S_11_ phase (Li_2_S:P_2_S_5_ = 7:3).^[^ ^1^, ^16^ ^]^ Thus, we considered four different environments for the PS_4_^3−^ units. To be specific, the PS_4_^3−^ units can exist in amorphous (disordered), Li_7_P_3_S_11_, *β*‐Li_3_PS_4_, and the argyrodite‐type (including BH_4_^−^) phases. For the *x* = 0 sample (after the first‐step milling), the argyrodite‐type phase can be excluded. By considering the previously reported results and our deconvolution process, the PS_4_^3−^ units in the amorphous, Li_7_P_3_S_11_, and *β*‐Li_3_PS_4_ phases were assigned to ≈83.4,^[^ ^16a^ ^]^ 85.5,^[^ ^23^ ^]^ and 86.1 ppm,^[^ ^16^ ^]^ respectively. For the *x* = 0.50–0.82 samples, a sub‐profile was mainly observed at ≈89.5 ppm, which can be reasonably attributed to the PS_4_^3−^ units in the argyrodite‐type phase containing the BH_4_^−^ anions formed after the second‐step milling. Additionally, the sub‐profile centered at ≈90.5 ppm corresponded to the P_2_S_7_^4−^ units.^[^ ^23^ ^]^

In the *x* = 0 sample, the abundance ratios of the PS_4_^3−^ units in the amorphous, Li_7_P_3_S_11_, and *β*‐Li_3_PS_4_ phases were 12.2%, 5.6%, and 77.1%, respectively. The PS_4_^3−^ units mainly existed in the *β*‐Li_3_PS_4_ phase, and this observation was corroborated by the XRD results as well. With increasing the amount of LiBH_4_, the areal proportion of the PS_4_^3−^ units in the argyrodite‐type phase appeared and increased. The ratios were 17.1%, 79.4%, and 91.6% in the *x* = 0.33, 0.50, and 0.54 samples, respectively, and they remained almost the same with the increasing *x* value up to *x* = 0.82. As observed in the XRD and Raman results, the formation of the argyrodite‐type phase was saturated at *x* = 0.54, well agreeing with the ionic conductivity values of the (1−*x*)Li_3_PS_4_·2*x*LiBH_4_ samples (Table 1). The ionic conductivity of the *x* = 0 sample was only 6.6 × 10^−4^ S cm^−1^, which increased to 1.4, 7.0, and 11 mS cm^−1^ for the *x* = 0.33, 0.50, and 0.54 samples, respectively. This rapid increase in the ionic conductivity can be well explained by the fact that the proportion of the argyrodite‐type phase substituted by the BH_4_^−^ anions increases with the increasing LiBH_4_ amount. It should be noted that the amount of the argyrodite‐type phase dominantly determines the ionic conductivity. Conversely, the abundance ratio of the PS_4_^3−^ units in the *β*‐Li_3_PS_4_ phase drastically decreased from *x* = 0 to *x* = 0.33 and 0.50, because the *β*‐Li_3_PS_4_ phase reacted with LiBH_4_ to form the argyrodite‐type phase. The proportions of the other phases, such as the PS_4_^3−^ units, in the amorphous and the Li_7_P_3_S_11_ phases, and the P_2_S_7_^4−^ units exhibited a gradual decreasing trend with the formation reactions of the argyrodite‐type phase. Above *x* = 0.54, the areal ratios of all kinds of thiophosphate units remained almost unchanged, because the formation of the argyrodite‐type phase was saturated, and the amount of unreacted LiBH_4_ increased with the increasing *x* value up to 0.82. Therefore, the ionic conductivity of the samples decreased. From the quantitative analysis results of the ^31^P MAS NMR spectra, it can be concluded that the composition with *x* = 0.54 is the maximum to form the BH_4_‐substituted argyrodite‐type phase, and thus, it exhibited the highest ionic conductivity among the prepared samples. In an aspect of the units as a by‐product, interestingly, the areal proportion of the P_2_S_7_^4−^ units, which formed as by‐products, decreased with the increasing LiBH_4_ concentration. One plausible explanation is that the addition of LiBH_4_ may break the bridging sulfur in the P_2_S_7_^4−^ units into PS_4_^3−^ units. Subsequently, the newly formed PS_4_^3−^ units may incorporate into the argyrodite‐type phase.

**Figure 5:**
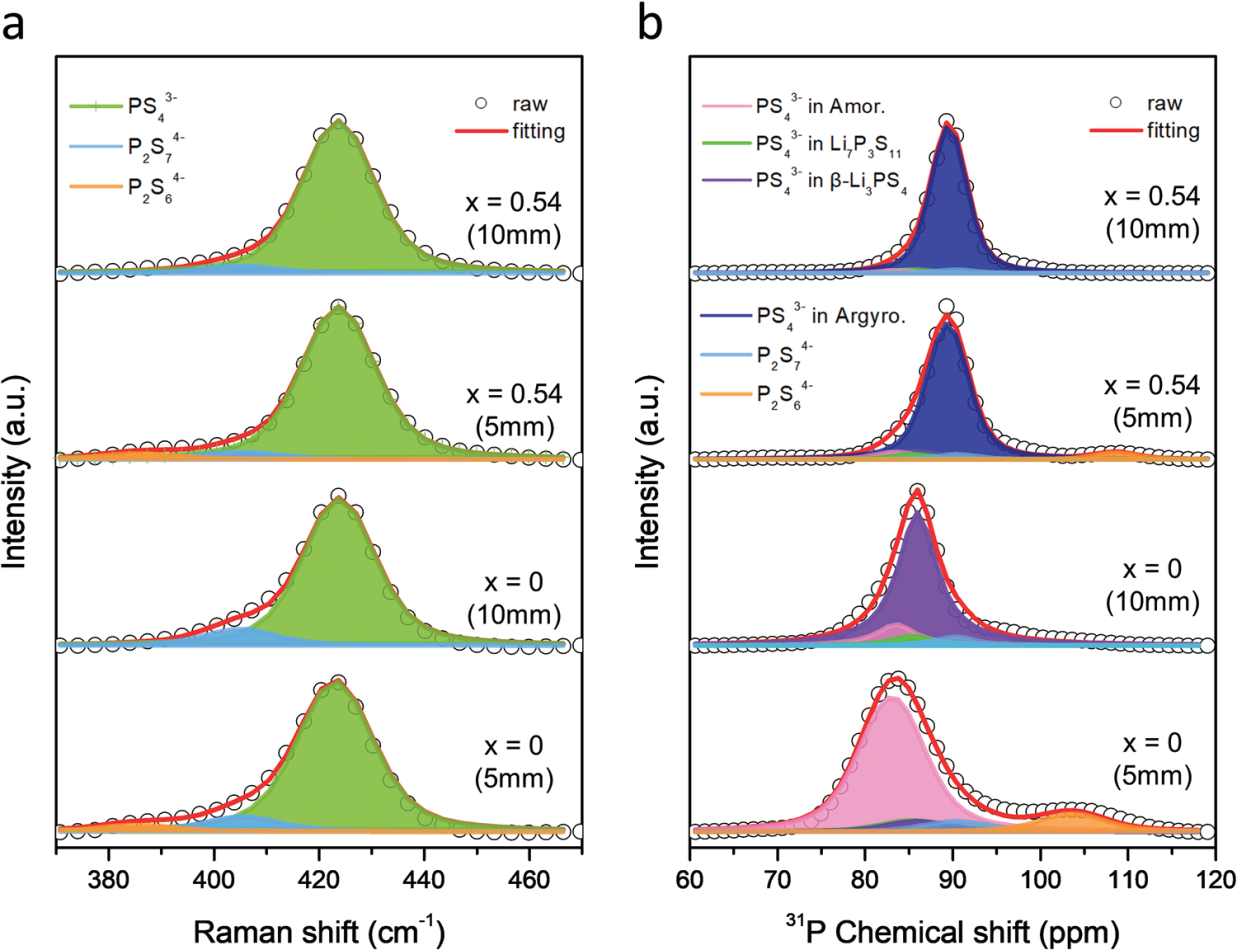
a) Raman spectra with deconvoluted profiles and b) ^31^P solid‐state MAS NMR spectra with deconvoluted profiles of the *x* = 0 (5mm), *x* = 0 (10mm), *x* = 0.54 (5mm), and *x* = 0.54 (10mm) samples.

Next, to reveal the changes in the thiophosphate local structure under different milling conditions and its influence on the ionic conductivity, we compared the Raman and ^31^P MAS NMR spectra with deconvoluted sub‐profiles of the “5mm” and “10mm” samples at the compositions of *x* = 0 and 0.54, and the results are shown in **Figure** **5** and **Table** **3**. The Raman spectra of the samples are presented in Figure 5a. As described above, the sub‐profiles centered at 423, 405, and 385 cm^−1^ can be attributed to the PS_4_^3−^, P_2_S_7_^4−^, and P_2_S_6_^4−^ anion units, respectively. For both the “10mm” samples prepared in this study, the peaks of P_2_S_6_^4−^ at 385 cm^−1^ were not detected. However, substantial amounts of P_2_S_6_^4−^ (3.9% and 3.5% for *x* = 0 and 0.54, respectively) were found in the “5mm” sample. As mentioned before, the milling energy in the “5mm” condition was not sufficient to form the *β*‐Li_3_PS_4_ phase in the first‐step milling, and thus, the nonstoichiometric P_2_S_6_^4−^ units were possibly produced as a by‐product via the side reaction. The units still remained after the second‐step milling because of their high stability,^[^ ^16a^ ^]^ resulting in the reduction of the proportion of the PS_4_^3−^ units in the samples.

**Table 3:** Abundance ratios of the thiophosphate units calculated from deconvolution data of the Raman spectra (Voigt product function) and ^31^P solid‐state MAS NMR spectra (Voigt and Lorentzian product function) of the *x* = 0 (5mm), *x* = 0 (10mm), *x* = 0.54 (5mm), and *x* = 0.54 (10mm) samples

	Raman spectra				^31^P solid‐state MAS NMR spectra						
*x*	Peak indexing	PS_4_^3−^ units	P_2_S_7_^4−^ units	P_2_S_6_^4−^ units	Peak indexing	PS_4_^3−^ units in amorphous phase	PS_4_^3−^ units in Li_7_P_3_S_11_	PS_4_^3−^ units in *β*‐Li_3_PS_4_	PS_4_^3−^ units in Argyrodite phase	P_2_S_7_^4−^ units	P_2_S_6_^4−^ units
0 (5mm)	Peak (cm^−1^)	423	405.2	385.7	Peak (ppm)	83.4	85.4	86.1	‐	90.4	106.1
	Area (%)	87.4	8.7	3.9	Area (%)	75.1	6	5.6	‐	5.7	8.6
0 (10mm)	Peak (cm^−1^)	422.9	404.9	‐	Peak (ppm)	83.5	85.4	86	‐	90.4	‐
	Area (%)	90.9	9.1	‐	Area (%)	12.2	5.6	77.1	‐	5.1	‐
0.54 (5mm)	Peak (cm^−1^)	423.6	405.1	385.8	Peak (ppm)	83.5	85.5	86	89.4	90.4	108.8
	Area (%)	92.1	4.4	3.5	Area (%)	4.4	3	4.8	80.8	3.1	3.9
0.54 (10mm)	Peak (cm^−1^)	423.5	405.1	‐	Peak (ppm)	83.5	85.4	86.1	89.6	90.4	‐
	Area (%)	95.4	4.6	‐	Area (%)	1.4	2.3	2.6	91.6	2.1	‐

Figure 5b shows the ^31^P MAS NMR spectra with deconvoluted sub‐profiles of the samples. After the first‐step milling, the abundance ratio of the PS_4_^3−^ units in the amorphous phase in the *x* = 0 (5mm) sample was 75.1% (at 83.4 ppm), which was the highest, while that of the PS_4_^3−^ units in the *β*‐Li_3_PS_4_ phase in the *x* = 0 (10mm) sample was 77.1% (at 86.1 ppm). These results well correspond to the peak assignment in the XRD analysis. As seen in the Raman spectra, the resonance signals for the P_2_S_6_^4−^ units were detected at 106.1 ppm in the *x* = 0 (5mm) sample, while they were not present in the spectra of the *x* = 0 (10mm) sample. After the second‐step milling, the PS_4_^3−^ units in the argyrodite‐type phase were the major components in both the cases. The areal ratios were 80.8% and 91.6% in the *x* = 0.54 (5mm) and *x* = 0.54 (10mm) samples, respectively. The *x* = 0.54 (10mm) sample exhibited a higher proportion of the PS_4_^3−^ units in the argyrodite‐type phase and lower proportions of the others such as the PS_4_^3−^ units in the amorphous, Li_7_P_3_S_11_, and *β*‐Li_3_PS_4_ phases, P_2_S_7_^4−^, and P_2_S_6_^4−^ units. As observed in the Raman analysis results, the presence of P_2_S_6_^4−^ units after the first‐step milling in the *x* = 0 (5mm) sample led to the lower proportion of the PS_4_^3−^ units and especially the lower proportion of the argyrodite‐type phase after the addition of LiBH_4_. As a result, the ionic conductivity of the *x* = 0.54 (5mm) sample was found to be much lower than that of the *x* = 0.54 (10mm) sample. Therefore, to achieve a high ionic conductivity in the BH_4_^−^‐containing thiophosphate electrolytes, it is important to suppress the formation of the P_2_S_6_^4−^ units and increase the proportion of the argyrodite‐type phase.

**Figure 6:**
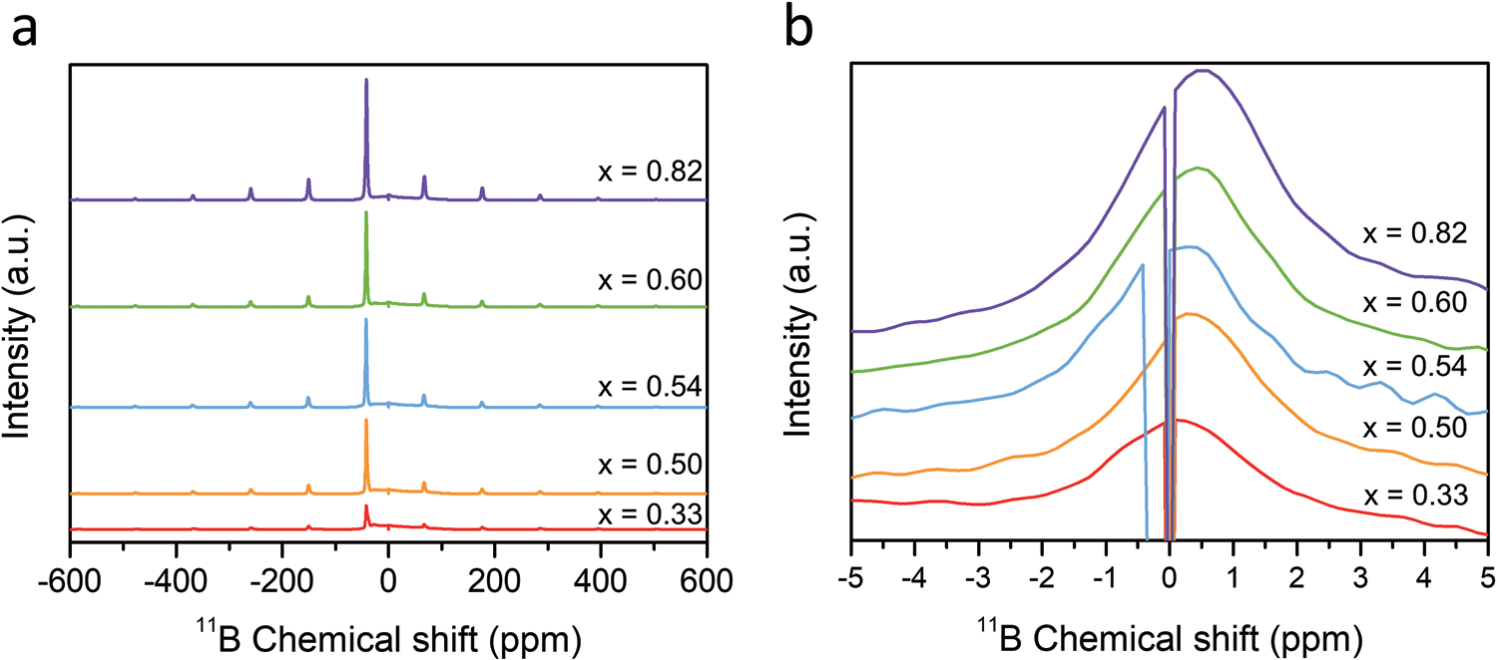
a) ^11^B solid‐state MAS NMR spectra of the as‐prepared solid electrolyte samples with the composition of (1−*x*)Li_3_PS_4_·2*x*LiBH_4_ and b) enlarged spectra from −5 to 5 ppm.

In the Raman and ^31^P MAS NMR analyses results, the formation of nonstoichiometric thiophosphate units, such as P_2_S_7_^4−^ and P_2_S_6_^4−^, was evident, implying the possible formation of boron–sulfur compounds through reactions between the thiophosphate units and LiBH_4_. To investigate this phenomenon, the ^11^B solid‐state MAS NMR spectra of the prepared solid electrolyte samples with the composition of (1−*x*)Li_3_PS_4_·2*x*LiBH_4_ were analyzed, and the results are shown in **Figure** **6**a. All the samples show a main peak at −42 ppm, which can be assigned to the resonance of the BH_4_^−^ anions.^[^ ^24^ ^]^ With increasing the amount of LiBH_4_, the peak intensity naturally increased. In addition, some distinctive minor peaks between −5 and 5 ppm were observed.

These peaks can be identified by analyzing the enlarged spectra presented in Figure 6b. The distinctive minor peak between 0 and 1 ppm can be attributed to the boron–sulfur compounds, such as BS_4_ and B_2_S_3_ compounds.^[^ ^25^ ^]^ As the amount of LiBH_4_ was increased, the observed peak intensity gradually increased. From Raman analysis results, we already identified the S—S bond breaking with the addition of LiBH_4_, indicating the possible existence of free sulfur. Therefore, one possible mechanism is that the free sulfur can react with the separated borohydrides, which originate from the decomposition of LiBH_4_,^[^ ^24c^ ^]^ to form the boron–sulfur compounds. The presence of the boron–sulfur compounds also suggests that some proportion of LiBH_4_ cannot form the argyrodite‐type phase by reacting with the PS_4_^3−^ anions and may decompose. In the previous reports, a halogen substitution ratio to the S sites of maximum 2 was reported in Li argyrodite phases (Li_7−_ *_y_* PS_6−_ *_y_* X*_y_*, X = Cl, Br, and I, *y* ≤ 2).^[^ ^20^, ^26^ ^]^ In this study, the highest ionic conductivity was observed at the composition of *x* = 0.54 (Li_3_PS_4_:LiBH_4_ = 1:2.3 in molar ratio), in which case the maximum amount of the argyrodite‐type phase containing the BH_4_^−^ anions was formed. Here, the molar ratio of LiBH_4_ to Li_3_PS_4_ was more than 2. This can be explained by the fact that some amount of LiBH_4_ decomposes during milling process and the separated borohydrides react with the free sulfur. The ^11^B MAS NMR spectra of the *x* = 0.54 (5mm) and *x* = 0.54 (10mm) samples are compared in Figure S13 (Supporting Information, please see the related text). The ^7^Li solid‐state MAS NMR spectra of the prepared solid electrolyte samples with the composition of (1−*x*)Li_3_PS_4_·2*x*LiBH_4_ are shown in Figures S14 and S15 (Supporting Information, please see the related text). The results are well consistent with those of the XRD and DSC.

**Figure 7:**
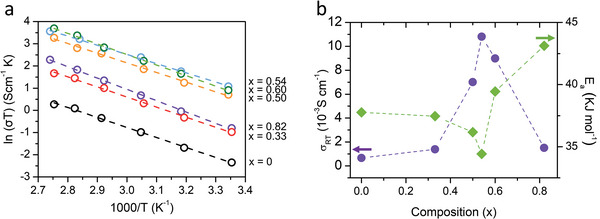
a) Temperature dependence of ionic conductivities and b) ionic conductivity at 25 °C and activation energy variation of the prepared solid electrolyte samples with the composition of (1−*x*)Li_3_PS_4_·2*x*LiBH_4_.

**Figure** **7**a shows the temperature dependence of the conductivity of the as‐prepared solid electrolytes with a composition of (1−*x*)Li_3_PS_4_·2*x*LiBH_4_. The conductivity curves were fitted with Arrhenius's law, as described in Equation (1)

(1) $$\sigma T=A\exp\left(-\frac{E_{a}}{RT}\right)$$

where *E*_a_ is the activation energy, *A* is the pre‐exponential factor, *σ* is the ionic conductivity, *T* is the absolute temperature, and *R* is the gas constant. The ionic conductivity at room temperature (298 K) and the activation energy of the prepared solid electrolyte samples are presented in Figure 7b. Additionally, the activation energy values are listed in Table S1 in the Supporting Information. The *x* = 0.54 sample showed the highest ionic conductivity and the lowest activation energy (34.4 kJ mol^−1^). As mentioned before, the ionic conductivity increased, whereas the activation energy decreased with increasing the amount of LiBH_4_ up to *x* = 0.54, beyond which the trends were reversed. Specifically, the *x* = 0.82 sample showed a much higher activation energy (43.1 kJ mol^−1^), because it contained a large amount of unreacted LiBH_4_.^[^ ^6a^ ^]^ The highest ionic conductivity and lowest activation energy of the *x* = 0.54 sample were obtained because of the saturation of the argyrodite‐type phase containing including BH^4−^. These results were well supported by the Raman and NMR analyses.

Figure S16 and Table S2 in the Supporting Information show the temperature dependence of conductivity of the *x* = 0 and 0.54 (5mm) samples. The *x* = 0 and 0.54 (5mm) samples exhibited lower ionic conductivities and higher activation energies than did the *x* = 0 and 0.54 (10mm) samples. The ^31^P MAS NMR results showed that the *x* = 0 (5mm) sample contained a higher proportion of the amorphous Li_3_PS_4_ phase and a lower proportion of the *β*‐Li_3_PS_4_ phase, compared with the *x* = 0 (10mm) sample. The ionic conductivity of the *β*‐Li_3_PS_4_ phase was higher than that of the amorphous Li_3_PS_4_ phase,^[^ ^27^ ^]^ and this explains the ionic conductivity difference of the *x* = 0 samples prepared under different milling conditions. The ionic conductivity of the *x* = 0.54 samples was well explained by the Raman and ^31^P MAS NMR results.

**Figure 8:**
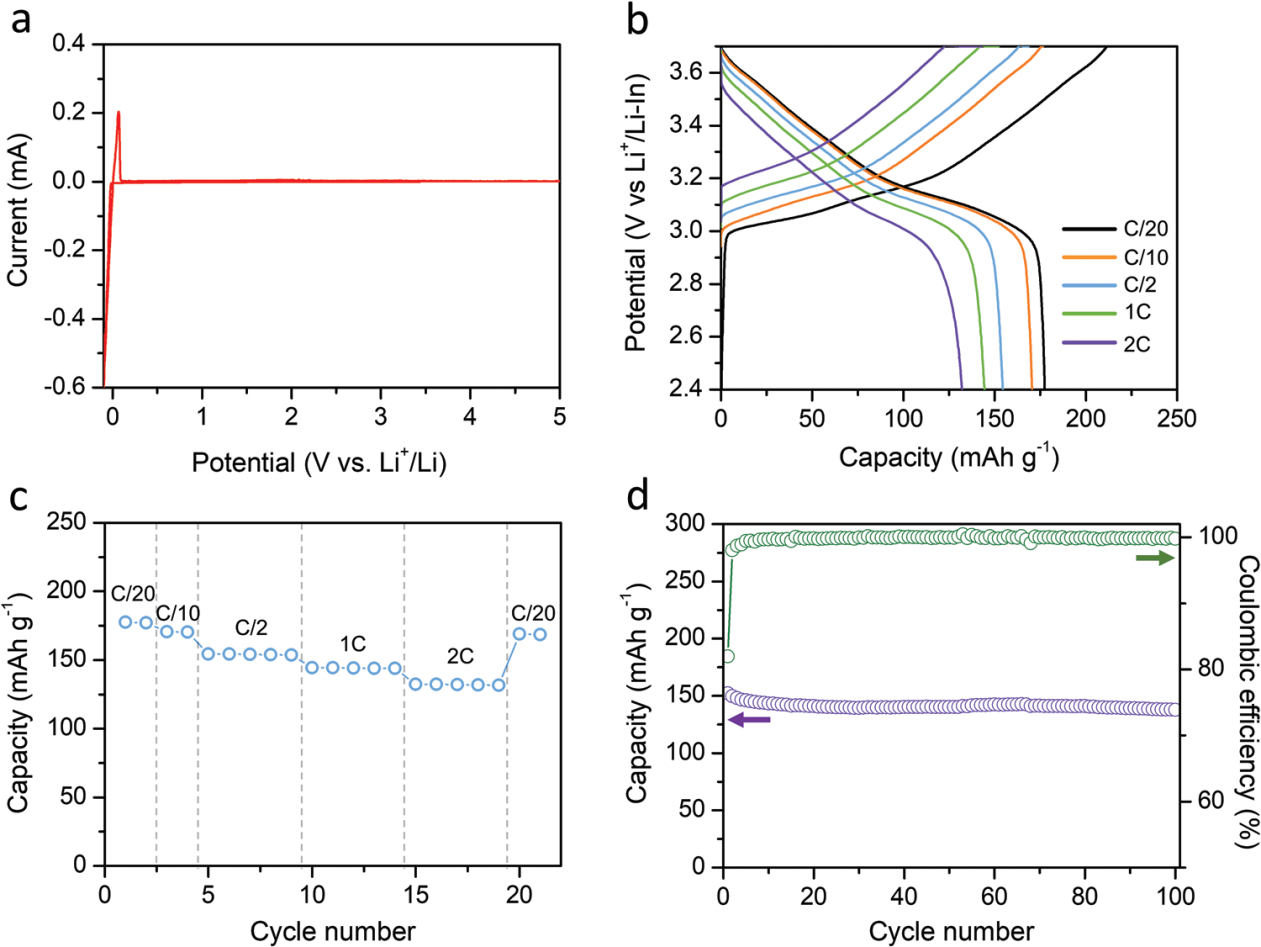
a) CV curves of the Li/solid electrolyte/SS cell, b) voltage profiles, and c) rate performance, d) cycling performance at 0.5 C of the ASSB incorporating the *x* = 0.54 (10mm) sample as the solid electrolyte material.

**Figure** **8** shows the electrochemical properties of the cells containing the *x* = 0.54 (10mm) sample as the solid electrolyte material. Figure 8a shows the cyclic voltammograms (CV) of the Li/solid electrolyte/stainless steel (SS) cell measured at 1 mV s^−1^. The cathodic and anodic peaks at −0.09 and 0.06 V (vs Li^+^/Li) were observed, respectively, which can be attributed to the deposition and dissolution of Li. Except for these reactions, there was no reaction up to 5.0 V (vs Li^+^/Li), indicating that the solid electrolyte samples had a wide electrochemical stability window up to 5.0 V. The solid electrolyte was evaluated also in a Li symmetric cell (Figure S17, Supporting Information). Figure 8b–d presents the electrochemical performance of the ASSB containing the Li/In anode and LiNi_0.7_Mn_0.15_Co_0.15_O_2_ cathode. The specific capacity was calculated based on the mass loading of the cathode. The voltage profiles at various C rates are shown in Figure 8b. The first charge and discharge capacities of the cathode at 0.05 C were measured to be 212.3 and 177.5 mAh g^−1^, respectively, with an initial Coulombic efficiency of 83.6%. The shapes of the curves are typical for layered cathodes. Figure 8c shows the rate performance of the cell. The discharge capacity at a high rate of 2 C was 132.3 mAh g^−1^. The capacity was well maintained at the high rate, indicating that the solid electrolyte works well at higher rates. The cycle performance was assessed at 0.5 C, and the result is presented in Figure 8d. The reversible capacity was well retained up to 100 cycles without degradation. This implies that the prepared solid electrolyte can be compatible with all‐solid‐state full cells without noticeable side reactions during long charge–discharge cycles.

## 3 Conclusion 

In this study, we prepared Li thiophosphate solid electrolytes containing BH_4_^−^ ions via a simple and fast milling method without heat treatment. The synthesized sulfide material exhibited a high ionic conductivity of up to 11 mS cm^−1^ at 25 °C. By adjusting the amount of LiBH_4_ inserted into Li_3_PS_4_ and the milling conditions, the thiophosphate local structure of the prepared samples was found to exhibit different states. The synthesized materials were analyzed by XRD, Raman spectroscopy, and solid‐state MAS NMR methods. Based on the quantitative and qualitative analyses results, it can be concluded that the PS_4_^3−^ units can exist in amorphous (disordered), Li_7_P_3_S_11_, *β*‐Li_3_PS_4_, and the argyrodite‐type (including BH_4_^−^) phases. The maximum conductivity was obtained when the proportion of the argyrodite‐type phase in the milled samples was the highest, and the PS_4_^3−^ anion units mainly existed in the argyrodite‐type phase. Conversely, the formation of nonstoichiometric thiophosphate units, such as P_2_S_7_^4−^ and P_2_S_6_^4−^, had an adverse effect on the ionic conductivity. Therefore, it is concluded that the formation of the argyrodite‐type phase consisting of the PS_4_^3−^ anion units without the nonstoichiometric thiophosphate units is essential to realize borohydride‐substituted thiophosphates with high ionic conductivities. The solid electrolyte samples were incorporated in a Li‐In/NCM all‐solid‐state cell to analyze their performance, and it was found that these cells exhibited superior cycle and rate performances.

## 4 Experimental and Computational Section

### Materials Synthesis

In this study, (1−*x*)Li_3_PS_4_·2*x*LiBH_4_ solid electrolytes were prepared via a simple and fast two‐step mechanical milling method. The Li_2_S (Alfa Aesar, 200 mesh, 99.9%), P_2_S_5_ (Sigma Aldrich, 99%), and LiBH_4_ (Acros Organic, 95%) powders were used as the starting materials without any treatment. First, the Li_2_S and P_2_S_5_ powders (3:1 by molar ratio) were placed into a zirconia vial with zirconia balls (10 mm diameter) and milled at 650 rpm for 1 h using a planetary mill (Retsch PM200). Then, the as‐obtained Li_2_S‐P_2_S_5_ composite and LiBH_4_ powders were ball‐milled using the same method for 1.5 h. The samples were processed in an Ar‐filled glovebox. The samples were prepared under different milling conditions (reference condition). The starting materials, mixing ratio, and milling machine were the same. The two‐step milling processes were performed at 213 rpm for 45 h (first step) and at 510 rpm for 15 h (second step) with different ball sizes (5 mm diameter), as reported previously.^[^ ^8^ ^]^

### Materials Characterization

XRD (Ultima IV, Rigaku) was used to characterize the crystal structure of the samples. DSC (Netzsch Polyma 214) was employed to examine the thermal properties. Raman spectroscopy was conducted using the LabRAM Aramis spectrometer (Horiba Jobin Yvon, 514 nm laser), and the acquired Raman spectra were deconvoluted using Voigt product functions. The solid‐state MAS NMR (400 MHz, Bruker ADVANCE III HD) spectra were obtained and deconvoluted using Lorentzian and Voigt product functions.

### Electrochemical Measurements and Fabrication of ASSBs

The ionic conductivity was measured with an SS (SUS440C)/solid electrolyte/SS cell using EIS (BioLogic VSP) in the frequency range from 1 MHz to 100 Hz at an amplitude of 100 mV and in the temperature range from 25 to 90 °C. To evaluate the electrochemical stability of the prepared solid electrolytes, CV was obtained. First, an appropriate amount of the electrolyte sample was pressed in a zirconia mold to form a pellet. Then, Li metal sheet was attached on the top side as a counter/reference electrode, and an SS plunger was used as the working electrode. The CV curves were obtained using a potentiostat (BioLogic VSP) at a scan rate of 1 mV s^−1^ in the range from −0.1 to 5.0 V at 25 °C. For the cell test, Li and In metal sheets were pressed and used as the anode. The cathode composites were prepared by mixing the active material (LiNi_0.7_Mn_0.15_Co_0.15_O_2_, 80 wt%), the prepared solid electrolyte (19 wt%), and a conducting agent (Super C, 1 wt%) in a vortex mixer. For the cell assembly, the prepared solid electrolyte powders (90 mg) were placed on a mold (diameter 10 mm) and then pressed at 312 MPa. Next, the cathode composites (9 mg) were positioned on the solid electrolyte pellet and compressed at 437 MPa. Finally, the Li/In foil anode was placed on the other side. The cells were assembled in an Ar‐filled glovebox and galvanostatically tested at various current densities (1.296 mA cm^−2^ = 1 C based on the cathode active material) at 30 °C. The rate and cycling tests of the ASSB were conducted with a battery cycler (Toyo Toscat‐3100) with voltage cutoff between 2.4 and 3.7 V (vs Li^+^/Li‐In).

### DFT Calculation

The crystal structure and energetics of Li_6_PS_5_X (X = Cl, Br, I and BH_4_) were simulated by DFT calculations. The DFT calculations were performed in the Perdew–Burke–Ernzerhof generalized‐gradient approximation,^[^ ^28^ ^]^ using Vienna Ab‐initio Simulation Package (VASP).^[^ ^29^ ^]^ A projector‐augmented wave (PAW) potential was used,^[^ ^30^ ^]^ and the PAW potentials employed were Li, P, S, Cl, Br, I, B, and H in the VASP pseudopotential library. A plane‐wave cutoff energy of 600 eV was used. The cubic unit cell containing four formula units (f.u.) of Li_6_PS_5_X was simulated, with a 3 × 3 × 3 *k*‐point grid, which ensures energy convergence of 1 meV f.u.^−1^. The cell volume and atomic positions were optimized until the force on each atom becomes less than 0.005 eV Å^−1^. The electronic charge on each atom was analyzed using the Bader charge analysis method.^[^ ^31^ ^]^

## Conflict of Interest

The authors declare no conflict of interest.

## Supporting information

Supporting Information

## Data Availability

The data that support the findings of this study are available from the corresponding author upon reasonable request.
