# Supplementary material for: Lithium Superionic Conduction in BH4‐Substituted Thiophosphate Solid Electrolytes
Source: Adv Sci (Weinh). 2022 Dec 11;10(5):2204942. doi: 10.1002/advs.202204942 (PMC9929267; doi:10.1002/advs.202204942)
Supplement: Supplementary file 1 — Supporting Information [file ADVS-10-2204942-s001.pdf]

ADVANCED SCIENCE
Open Access

Supporting Information

for *Adv. Sci.*, DOI 10.1002/advs.202204942

Lithium Superionic Conduction in BH_4-Substituted Thiophosphate Solid Electrolytes

Yong-Jin Jang, Hyungeun Seo, Young-Su Lee, Sora Kang, Woosuk Cho, Young Whan Cho and Jae-Hun Kim**

Supporting Information

Lithium Superionic Conduction in BH_4-Substituted Thiophosphate Solid Electrolytes

Yong-Jin Jang, Hyungeun Seo, Young-Su Lee, Sora Kang, Woosuk Cho, Young Whan Cho, and Jae-Hun Kim**

Y.-J. Jang, H. Seo, J.-H. Kim
School of Materials Science and Engineering, Kookmin University, Seoul 02707, Republic of Korea
E-mail: jaehunkim@kookmin.ac.kr

Y.-S. Lee, Y. W. Cho
Energy Materials Research Center, Korea Institute of Science and Technology, Seoul 02792, Republic of Korea
E-mail: oze@kookmin.ac.kr

S. Kang, W. Cho
Advanced Batteries Research Center, Korea Electronics Technology Institute, Seongnam, Gyeonggi 13509, Republic of Korea

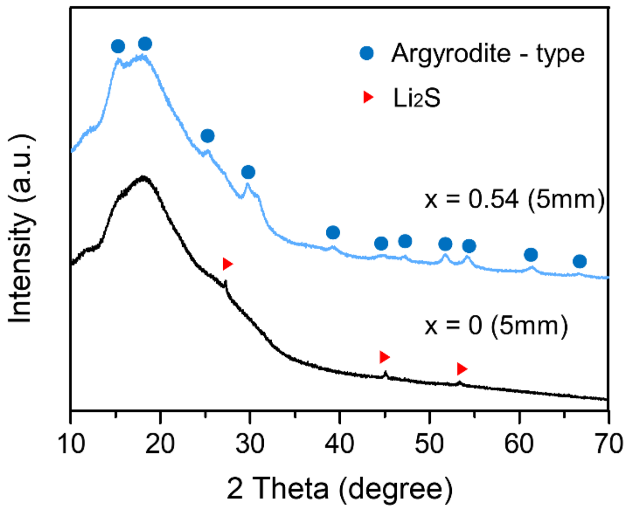

Figure S1. XRD patterns of the *x* = 0 (5mm) and *x* = 0.54 (5mm) samples prepared under the reference condition.

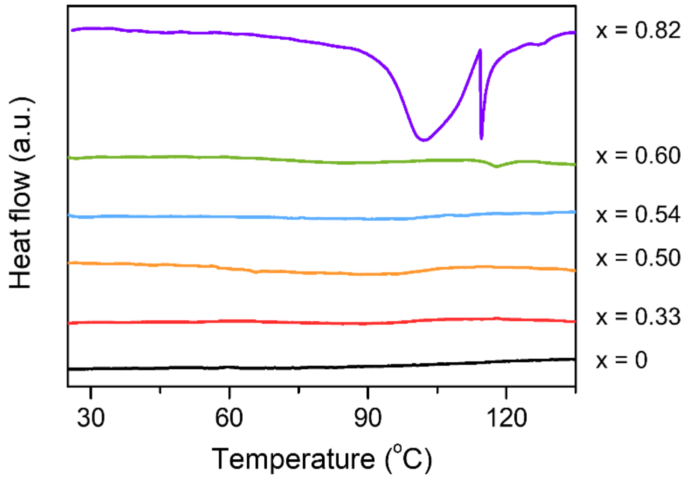

Figure S2. DSC curves of the prepared solid electrolyte samples with the composition of (1–x)$Li_3PS_4 \cdot 2xLiBH_4$. The broad endothermic peak just before the sharp peak of $LiBH_4$ phase transition is believed to be the thermal decomposition of the thiophosphates.

It is well known that pure $LiBH_4$ exhibits an endothermic peak near 113 °C, which is attributed to the phase transition from orthorhombic to hexagonal.[1,2] In the DSC profiles for the samples from $x = 0$ up to $x = 0.54$, there is no distinct endothermic peak. When x is 0.60, a small endothermic peak is observed at 117 °C. For the $x = 0.82$ sample, one can see a sharp endothermic peak at 113 °C, which is assigned to the phase transition of $LiBH_4$. These results indicate that only a negligible amount of unreacted $LiBH_4$ remained after the two-step milling until the composition reached $x = 0.54$. As x increased to 0.60, a small amount of $LiBH_4$ remained unreacted in the electrolyte sample. The $x = 0.82$ sample clearly shows a more distinctive and sharper endothermic peak than does the $x = 0.60$ sample, indicating that the $x = 0.82$ sample contained a higher proportion of the unreacted $LiBH_4$. From these results, it can be concluded that the $x = 0.54$ composition is optimum to incorporate $LiBH_4$ into the as-prepared Li_3PS_4 sample without leaving any unreacted amount.

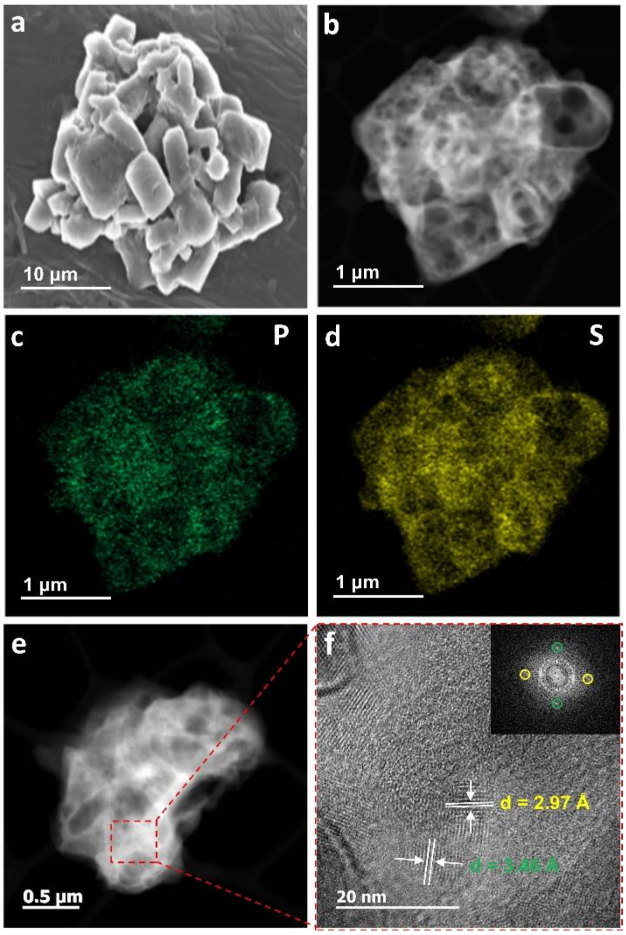

Figure S3. (a) FE-SEM, (b) TEM, (c,d) EDS elemental mapping images for P and S, (e) high-magnification TEM image, and (f) HR-TEM image with FFT pattern (inset) of the x = 0.54 (10mm) sample.

Figure S3a shows the field emission-scanning electron microscopy (FE-SEM, JSM 7401F, JEOL) image. Primary particles measuring a few micrometers aggregated to form secondary particles. Figure S3b exhibits the transmission electron microscopy (TEM, ARM-200F, JEOL) image with energy dispersive spectroscopy (EDS) elemental mapping results (Figure S3c and S3d). P and S elements were uniformly distributed in the entire particle. The high-magnification TEM image is shown in Figure S3e and its part was enlarged. The high-resolution TEM (HR-TEM) image with the fast Fourier transform (FFT) pattern of a selected area is presented in Figure S3f. The lattice spacing of the crystallites was measured to be 2.97 and 3.46 Å in different areas as indicated in the image by arrows.

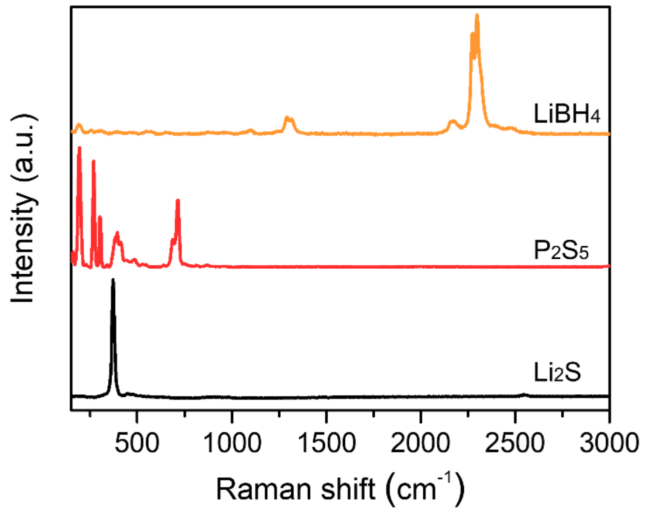

Figure S4. Raman spectra of the starting materials as references.

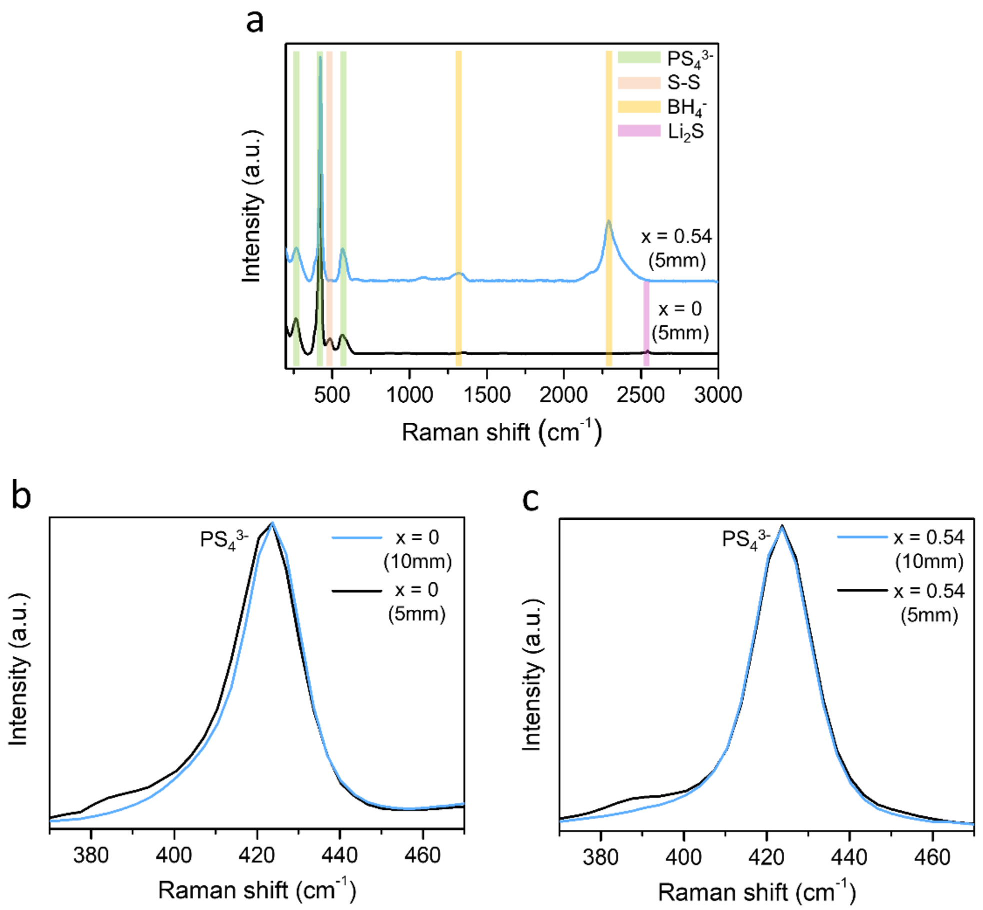

Figure S5. Raman spectra of (a) the x = 0 (5mm) and x = 0.54 (5mm) samples prepared under the reference condition, (b) the x = 0 (5mm) and x = 0 (10mm) samples, and (c) the x = 0.54 (5mm) and x = 0.54 (10mm) samples.

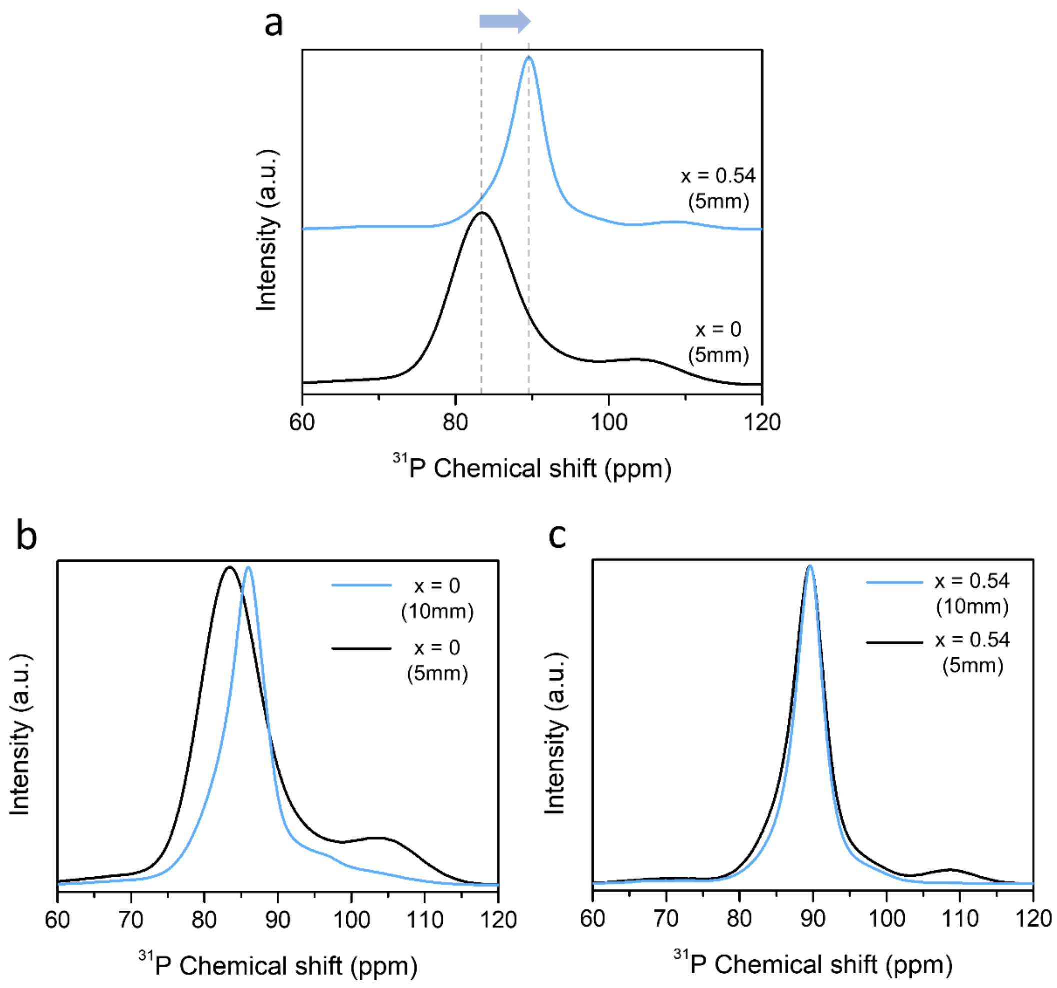

Figure S6. ^{31}P solid-state MAS NMR spectra of (a) the x = 0 (5mm) and x = 0.54 (5mm) samples prepared under the reference condition, (b) the x = 0 (5mm) and x = 0 (10mm) samples, and (c) the x = 0.54 (5mm) and x = 0.54 (10mm) samples.

Table S1. Activation energies of the prepared solid electrolyte samples with the composition of $(1 - x)Li_3PS_4 \cdot 2xLiBH_4$.

x	Composition	Activation energy ($kJ\ mol^{-1}$)
0	Li_3PS_4	37.8
0.33	$Li_4PS_4(BH_4)$	37.5
0.50	$Li_5PS_4(BH_4)_2$	36.2
0.54	$Li_{5.3}PS_4(BH_4)_{2.3}$	34.4
0.60	$Li_6PS_4(BH_4)_3$	39.4
0.82	$Li_{12}PS_4(BH_4)_9$	43.1

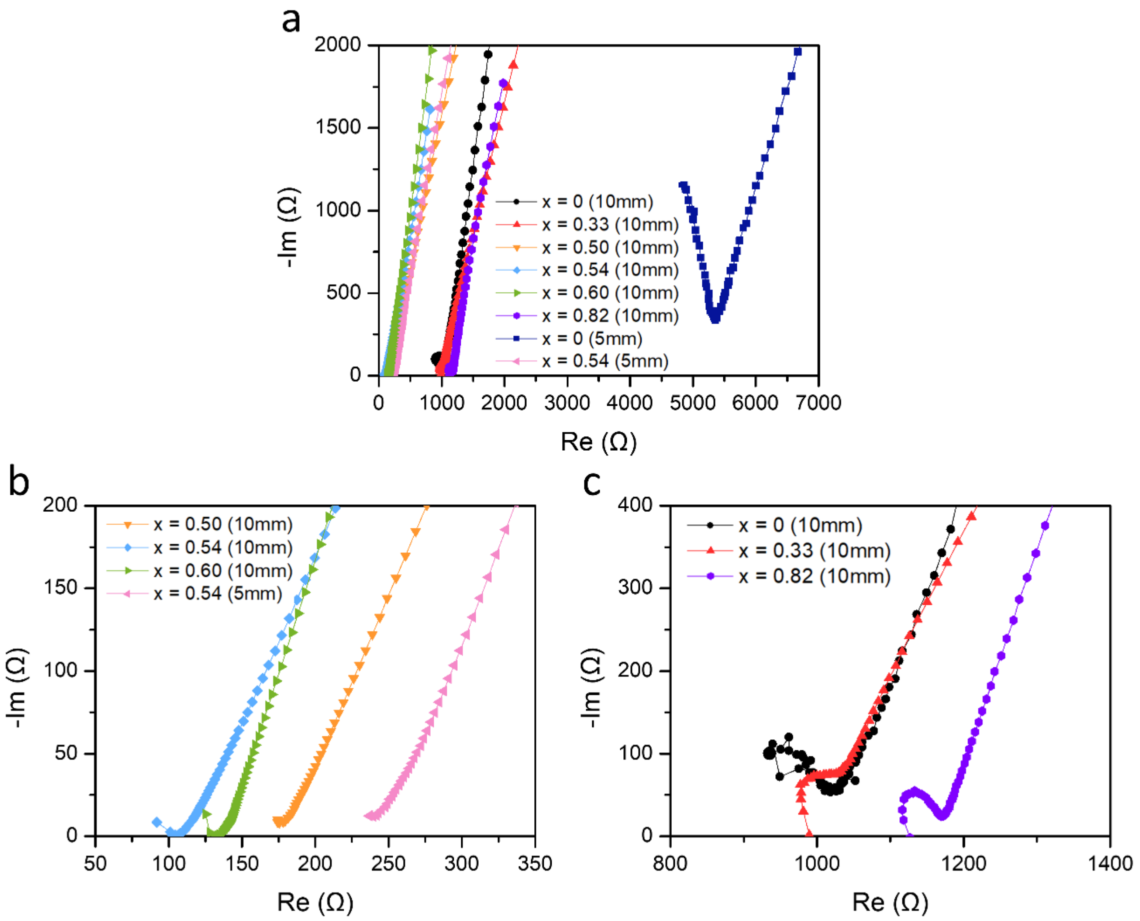

Figure S7. Nyquist plots of the cells containing the prepared solid electrolyte samples.

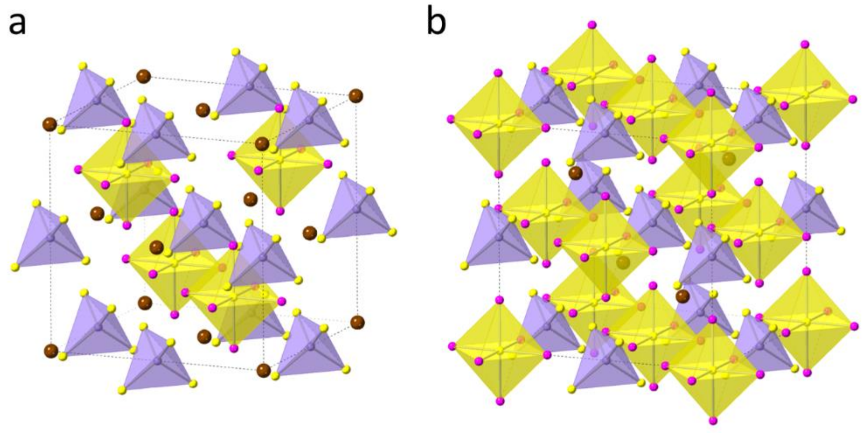

Figure S8. Crystal structure of (a) X@4*a* and (b) X@4*d*. PS_4 ions and S ions surrounded by six Li ions are illustrated as violet and yellow polyhedra, respectively. Li and X ions are drawn in magenta and brown, respectively.

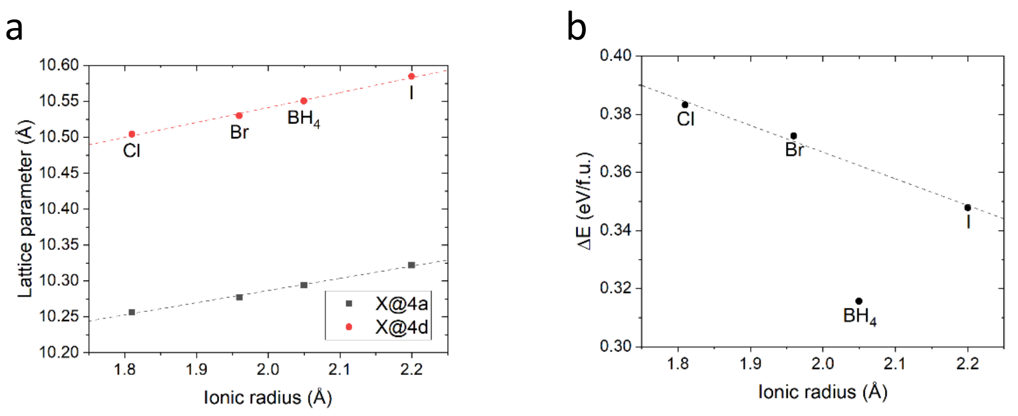

Figure S9. (a) Calculated lattice parameters of Li_6PS_5X and (b) energy difference per formula unit (f.u.) of Li_6PS_5X between the X@4*a* and X@4*d* structures, plotted against the ionic radius of X. The dashed lines are a linear regression of the data; the data of BH_4 was excluded in the linear regression in (b).

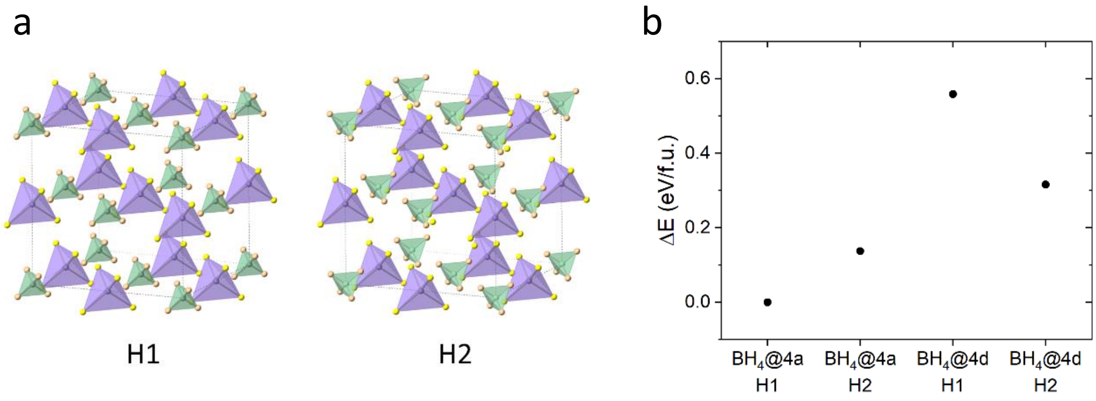

Figure S10. (a) Two types of BH_4 orientation labelled as H1 and H2. BH_4 and PS_4 ions are illustrated as green and violet polyhedera, respectively. Other atoms are not shown for clarity and (b) comparison of total energies of the four possible combinations among the (4*a*, 4*d*) site and the (H1, H2) orientation. The energy of the BH_4@4*a* structure having the H1 orientation is set to 0.

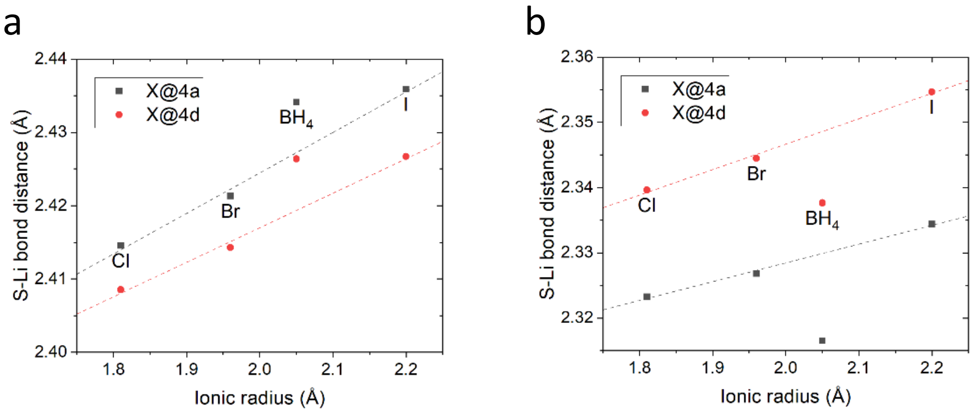

Figure S11. Bond distance between S and Li: (a) S at the 16*e* site and (b) S at the 4*d* (X@4*a*) or 4*a* (X@4*d*) site. The dashed lines are a linear regression of the data; the data of BH_4 was excluded in the linear regression.

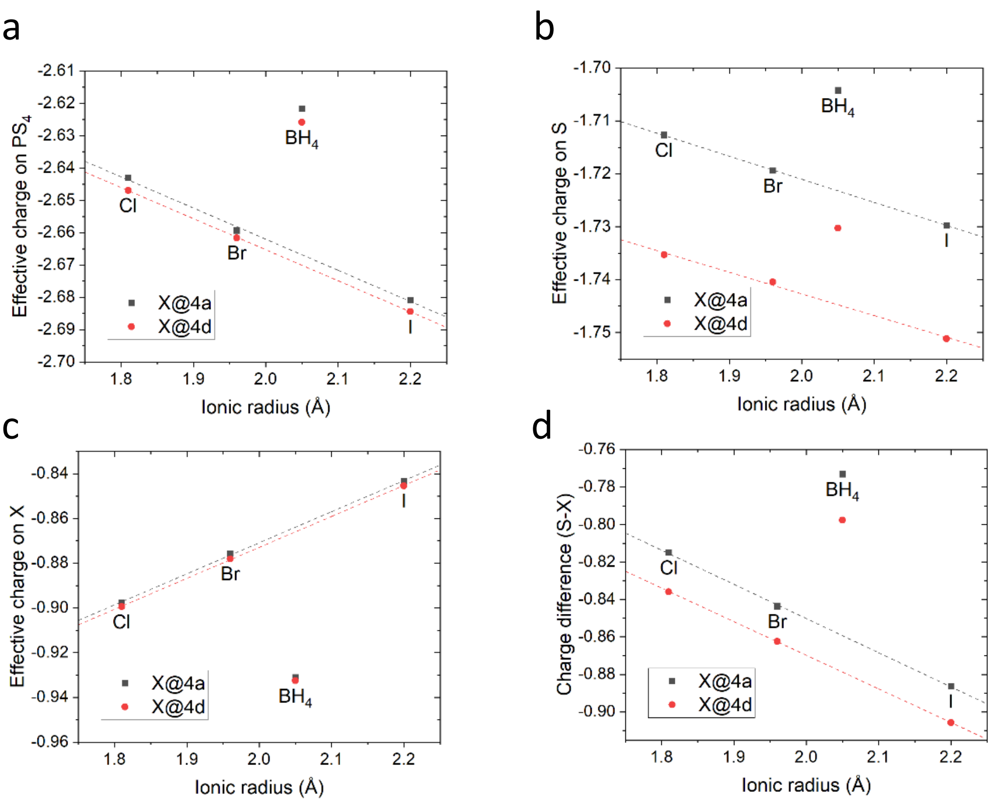

Figure S12. Effective charges on (a) PS_4, (b) S, (c) X and (d) the difference in effective charges between S and X. The dashed lines are a linear regression of the data; the data of BH_4 was excluded in the linear regression.

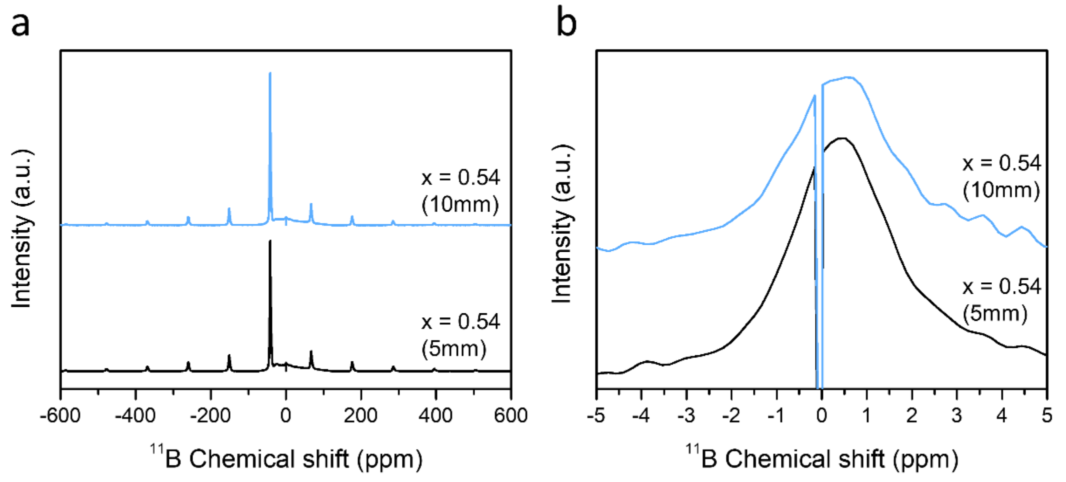

Figure S13. (a) ^{11}B solid-state NMR spectra of the *x* = 0.54 (5mm) and *x* = 0.54 (10mm) samples and (b) enlarged spectra from –5 ppm to 5 ppm.

The *x* = 0.54 (5mm) sample exhibits a lower intensity of the BH_4^- anion (at –42 ppm) peak and a higher intensity of the boron–sulfur compound (between 0 and 1 ppm) peak, compared to the *x* = 0.54 (10mm) sample. This implies that the decomposition of $LiBH_4$ and the formation of the boron–sulfur compounds in the *x* = 0.54 (5mm) sample were more pronounced compared to those in the *x* = 0.54 (10mm) sample. This agrees well with the ^{31}P MAS NMR results, which indicated that the *x* = 0 (5mm) sample contained more by-product units, such as $P_2S_7^{4-}$ or $P_2S_6^{4-}$, and the *x* = 0 (10mm) sample contained a higher proportion of the PS_4^{3-} units in the argyrodite-type phase.

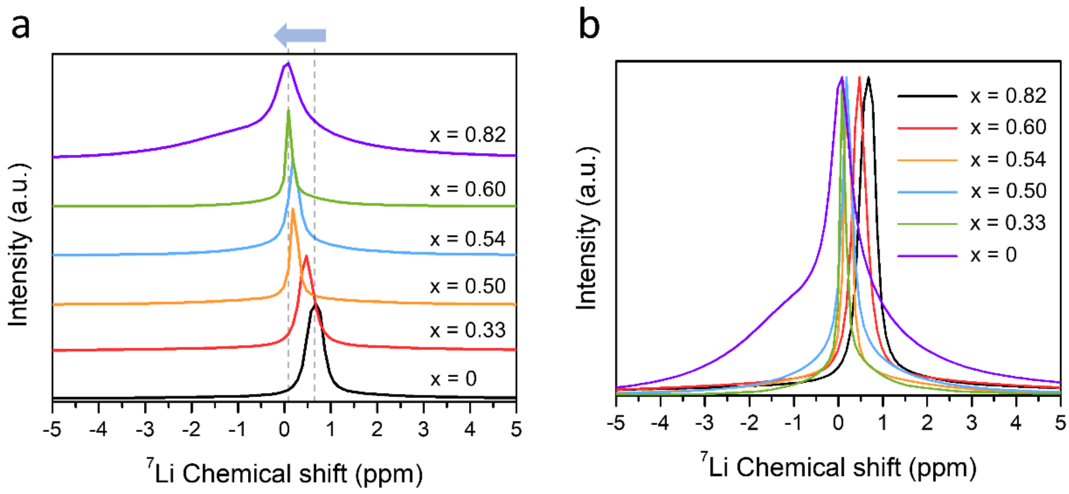

Figure S14. ^{7}Li solid-state MAS NMR spectra of the prepared solid electrolyte samples with the composition of (1–*x*)Li_3PS_4·2*x*$LiBH_4$.

The main peak of the *x* = 0 sample was observed at 0.67 ppm and can be ascribed to the PS_4^{3-} anion units in the β-Li_3PS_4 phase,[3,4] which were identified in the XRD analysis. With the increasing amount of $LiBH_4$ in the milled samples up to *x* = 0.60, the main peak position of each sample slightly shifted toward a lower frequency. For *x* > 0.60, the main peak position remained unchanged. This trend might be attributed to the effects of incorporating $LiBH_4$, because the main peak position of pure $LiBH_4$ is located at –1.2 ppm.[5] Moreover, the *x* = 0.82 sample shows a distinct shoulder around –1 ppm to –3 ppm, unlike the *x* = 0.60 sample, indicating that the *x* = 0.82 sample contained a large amount of unreacted $LiBH_4$. This result is well consistent with the XRD and DSC results.

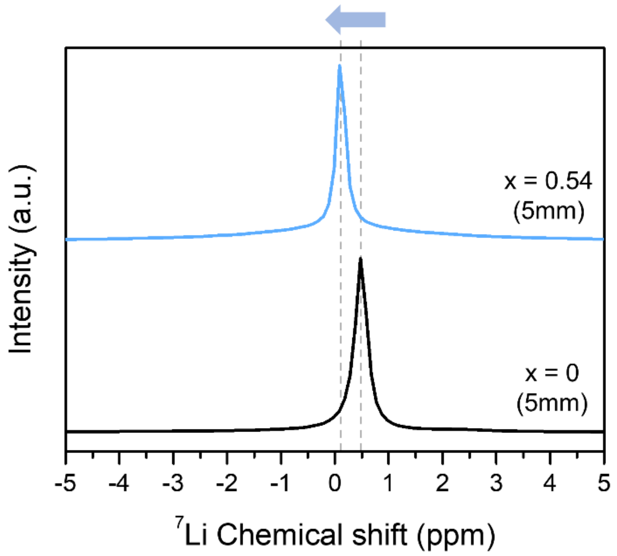

Figure S15. ^{7}Li solid-state MAS NMR spectra of the *x* = 0 (5mm) and *x* = 0.54 (5mm) samples prepared under the reference condition.

The main peak of the *x* = 0 (5mm) sample appeared at 0.48 ppm. This peak can be ascribed to the PS_4^{3-} anion units in the amorphous phase,[6] which were confirmed in the XRD analysis. With the addition of $LiBH_4$, the main peak position shifted toward a lower frequency and was located at 0.10 ppm.

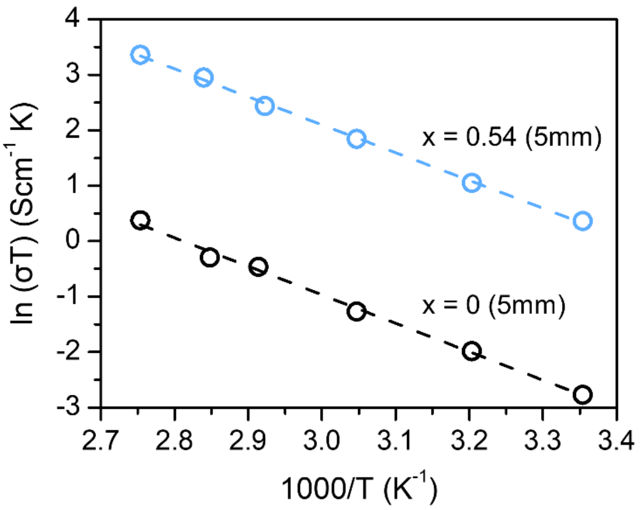

Figure S16. Temperature dependence of ionic conductivities of the x = 0 (5mm) and x = 0.54 (5mm) samples.

Table S2. Activation energies of the x = 0 (5mm) and x = 0.54 (5mm) samples.

x	Composition	Activation energy (kJ mol^{-1})
0	Li_3PS_4	42.7
0.54	$Li_{5.3}PS_4(BH_4)_{2.3}$	41.8

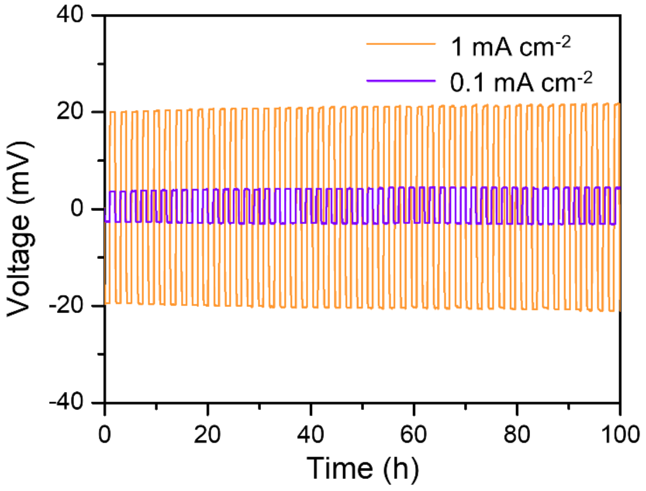

Figure S17. Voltage profiles of the Li metal/Solid electrolyte/Li metal symmetric cells.

The cells were tested at constant current densities of 0.1 and 1 mA cm^{-2} for Li deposition and dissolution (50 cycles). Each deposition or dissolution step was performed for 1 h. The overpotential for deposition and dissolution at the lower current density of 0.1 mA cm^{-2} was observed to be smaller (~3 mV) than that (~20 mV) at the higher current density of 1 mA cm^{-2} as expected. The overpotential at each current density was well maintained without changes up to 50 cycles. Traces of any side reactions were not detected.

References

[1] A. Sakuda, A. Yamauchi, S. Yubuchi, N. Kitamura, Y. Idemoto, A. Hayashi, M. Tatsumisago, ACS Omega **2018**, 3, 5453.

[2] M. Au, A. R. Jurgensen, W. A. Spencer, D. L. Anton, F. E. Pinkerton, S.-J. Hwang, C. Kim, R. C. Bowman, J. Phys. Chem. C **2008**, 112, 18661.

[3] M. Gobet, S. Greenbaum, G. Sahu, C. Liang, Chem. Mater. **2014**, 26, 3558

[4] E. C. Self, P.-H. Chien, L. F. O'Donnell, D. Morales, J. Liu, T. Brahmbhatt, S. Greenbaum, J. Nanda, Mater. Today Phys. **2021**, 21, 100478.

[5] Y. J. Choi, J. Lu, H. Y. Sohn, Z. Z. Fang, C. Kim, R. C. Bowman, S.-J. Hwang, J. Phys. Chem. C **2011**, 115, 6048.

[6] C. Dietrich, D. A. Weber, S. J. Sedlmaier, S. Indris, S. P. Culver, D. Walter, J. Janek, W. G. Zeier, J. Mater. Chem. A **2017**, 5, 18111.
